# A controlled randomized trial with a 12-week follow-up investigating the effects of medium-frequency neuromuscular electrical stimulation on pain, VMO thickness, and functionality in patients with knee osteoarthritis

**DOI:** 10.1186/s12891-024-07266-8

**Published:** 2024-02-20

**Authors:** Azar Moezy, Soheila Masoudi, Ahmad Nazari, Arezoo Abasi

**Affiliations:** 1 Department of Sports and Exercise Medicine, Iran University of Medical Sciences, Tehran, Iran; 2 Department of Sports and Exercise Medicine, School of Medicine, Iran University of Medical Sciences, Tehran, Iran; 3 Department of Health Information Management, School of Health Management and Information Sciences, Iran University of Medical Sciences, Tehran, Iran; 4 Department, School of Medicine, Hazrate Rasoole Akram Hospital, Iran University of Medical Sciences, Sattarkhan Ave, Niayesh St, Tehran, 14455613131 Iran

**Keywords:** NMES, Interferential Current, Knee Osteoarthritis, Pain, Function, Vastus medialis thickness

## Abstract

**Background:**

One of the major contributors to disability in Knee osteoarthritis (KOA) patients is weakness in the Quadriceps Femoris muscle. Neuromuscular electrical stimulation (NMES) has been used in rehabilitation for patients suffering from muscle weakness. Thus, the purpose of the study was to assess the effectiveness of NMES and exercise therapy, for improving pain, muscle weakness and function among patients with KOA.

**Methods:**

A randomized controlled trial was conducted with 75 female patients diagnosed with KOA. Participants were divided into three intervention groups: NMES-only, exercise therapy (Exs) alone, and a combination of NMES and exercise (NMES + Exs). All patients underwent 12 supervised treatment sessions, three times a week. Outcome measures included pain intensity measured by visual analog scale (VAS), knee flexion range of motion (FROM), thigh muscle girth (TG), thickness of the Vastus Medialis Oblique (VMO), timed up and go test (TUG), six-minute walk test (6MWT), and WOMAC scores. Statistical analyses (ANOVA and Kruskal–Wallis) methods were done to compare the amounts at the baseline, immediately after treatment and after 12 weeks.

**Results:**

The NMES group exhibited a significant reduction in pain at the 12-week follow-up compared to the other groups(*p* = 0.022). The NMES + Exs group showed better outcomes in terms of FROM, TG, and VMO thickness post-intervention (*p* < 0.0001, *p* < 0.004, *p* = 0.003, respectively) and at the 12-week follow-up (*p* < 0.0001, *p* < 0.0001, *p* < 0.0001, respectively). Additionally, NMES was superior in improving TUG and 6MWT post-intervention (*p* < 0.0001, *p* = 0.038, respectively) and during the follow-up assessments (*p* < 0.0001, *p* = 0.029, respectively). The NMES + Exs group achieved better WOMAC stiffness scores at both post-intervention and follow-up evaluations (*p* < 0.0001, *p* < 0.0001, respectively). Furthermore, at the 12-week follow-up, NMES + Exs group outperformed the others in WOMAC pain and function subscales (*p* = 0.003, *p* = 0.017, respectively), while the NMES group demonstrated better WOMAC total scores compared to the other groups (*p* = 0.007).

**Conclusion:**

The combination of NMES and exercise seems to be an efficient approach for managing KOA, as it enhances knee flexion range and TG, increases VMO thickness, and improves WOMAC scores. On the other hand, NMES alone was found to be effective in improving the physical function of KOA patients.

**Trial registration:**

IRCT20101228005486N7 (06–02-2020).

**Supplementary Information:**

The online version contains supplementary material available at 10.1186/s12891-024-07266-8.

## Introduction

Knee Osteoarthritis (KOA) is widely recognized as a significant public health issue [1] that leads to functional impairment and reduces people's quality of life globally, increasing with age and obesity [2]. The decline in neuromuscular function, including reduced joint proprioception, weakened muscles, an increased risk of falls, and muscle atrophy, is often associated with the onset of KOA [3]. These changes put extra-stress on the joint cartilage and contribute to the disease's progression [4].

Patients with KOA commonly experience Quadriceps Femoris (QF) muscle weakness, affecting 20% to 70% of cases [5]. This weakness is a major contributor to disability, particularly in individuals with severe knee pain reducing joint stabilization and shock absorption and accelerating osteoarthritic changes and cartilage degeneration [6, 7]. QF muscle atrophy is an early sign of KOA, mainly attributed to pain, swelling, and mechanoreceptor dysfunction, and significantly contributes to physical impairment [8].

Strengthening the QF muscles has been recognized as an essential part of non-surgical KOA management, providing significant therapeutic benefits [9–11].

Numerous studies have indicated that individuals with KOA frequently experience greater weakness in their Vastus Medialis Oblique(VMO) muscle, often presenting as an initial clinical symptom even before the onset of pain [4, 11].

Clinicians have various interventions to address quadriceps weakness in the rehabilitation process, and one of these is neuromuscular electrical stimulation (NMES) [12].

However, there is conflicting evidence regarding the optimal selection of NMES parameters, current frequency, electrode placement, and its overall effectiveness in improving quadriceps strength and function [13].Further research is needed to determine the most optimal application of NMES for this purpose [14].

Some studies have shown that NMES is superior to voluntary exercise in preventing muscle loss following a period of immobilization [13–16]. Limited evidence exists to support the effectiveness of NMES in restoring muscle mass in patients with KOA compared to conventional exercise therapy. A systematic review by Glaviano and Saliba pointed out deficiencies in current research on NMES, such as not using NMES in functional positions, inadequate attention to selected parameters, and suboptimal electrode placement. These limitations need to be addressed to better understand the potential benefits of NMES for patients with KOA [13]. Most previous studies have predominantly focused on using low-frequency electrical currents to improve muscle performance [12, 17–20]{Walls, 2010 #41}{Walls, 2010 #41}, with medium-frequency currents receiving less consideration. Nevertheless, medium-frequency currents such Interferential (IF) can more easily penetrate through the skin and muscles with minimal discomfort, rendering it a more bearable and acceptable alternative for patients [21].

Due to the scarcity of research on the application of IF currents in NMES for muscle strengthening, it is essential to investigate other treatment options, especially considering the high incidence of KOA and its associated physical impairments.

Furthermore, it is vital to acknowledge that some patients may be incapable of participating in exercise therapy due to severe pain and muscle weakness. Therefore, the purpose of this study is to assess the effectiveness of medium-frequency NMES in comparison to exercise therapy alone or a combination of both in managing pain, thigh muscle atrophy, the VMO muscle thickness, and physical function in women with primary KOA.

## Methods

### Design

The study was designed as a randomized controlled study with blinded assessors, in which patients were randomly assigned to one of three intervention groups: NMES only (NMES), exercise therapy alone (Exs) as a control group, or NMES combined with exercises (NMES + Exs).

### Patients

The study involved 75 female patients who were directed to Hazrat-e Rasool General Hospital, Iran University of Medical Sciences between 2020 and 2022.

This trial has received ethical approval from the Medical Ethics Committee of Iran University of Medical Sciences under the code IR.IUMS.FMD.REC.1398.351, in accordance with the most recent version of the Helsinki Declaration. Additionally, our study has been approved by the Iranian Registry of Clinical Trials with the ID of IRCT20101228005486N7. Prior to their participation, all patients included in the study provided written informed consent. Patients were permitted to withdraw from the study at any time without any condition if they chose not to continue. Furthermore, all patients were assured that their personal information would be treated confidentially and securely.

The patients had established cases of KOA that were confirmed based on the American College of Rheumatology criteria. The confirmation of the condition was determined by considering their clinical history, conducting physical examinations, and assessing radiographic changes.

The inclusion criteria were as follows: (1) X-ray stages II and III of KOA classified according to the criteria developed by Kellgren and Lawrence;(2) age between 50–75 years; (3) BMI ≤ 30; (4) Knee pain lasted for a minimum of 6 months with severity at least 3 on VAS scale during activities such as ascending and descending stairs, sitting, and squatting; (5) normal mental state.

The exclusion criteria were as follows: (1) previous knee and lower extremity surgery or injury; (2) neuromuscular disease; (3) bone implants; (4) history of lower extremity fractures within the last 6 months; (5) cancerous tumors; (6) history of acute traumatic injuries or chronic disease and any other conditions affecting the study; (7) participating in sports programs and physical therapy in the recent three months; (8) intra- knee injection in the last six months; (9) usage of opioid analgesics or systemic corticosteroids within the last 4 weeks, (10) inability to do exercise due to extreme pain or other limitations, (11) unwillingness to participate in the study; and (12) incomplete assessment/treatment programs.

### Sample size

The sample size for each group was determined to be 20, taking into account a significance level (α) of 0.05 and a type two error rate (ß) of 0.20 (power = 80%). This sample size was chosen to detect a difference of two points on the pain scale (VAS) [6]. However, to compensate for potential dropouts, the sample size was increased to 25 patients per group.

### Study settings

Interested patients were assessed by a specialist to confirm their eligibility for the study. Subsequently, the patients underwent a baseline assessment including VAS for pain, the range of active knee flexion (FROM), thigh girth (TG) measurement, ultrasonographic measurement of VMO thickness, 6-min walk (6MWT), timed up and go (TUG) tests, and WOMAC questionnaire. The outcome measures were administered at three time points: at the baseline or pre-intervention and post-intervention (at 4 weeks) and after 12-week follow-up.

### Randomized allocation

**Fig. 1:**
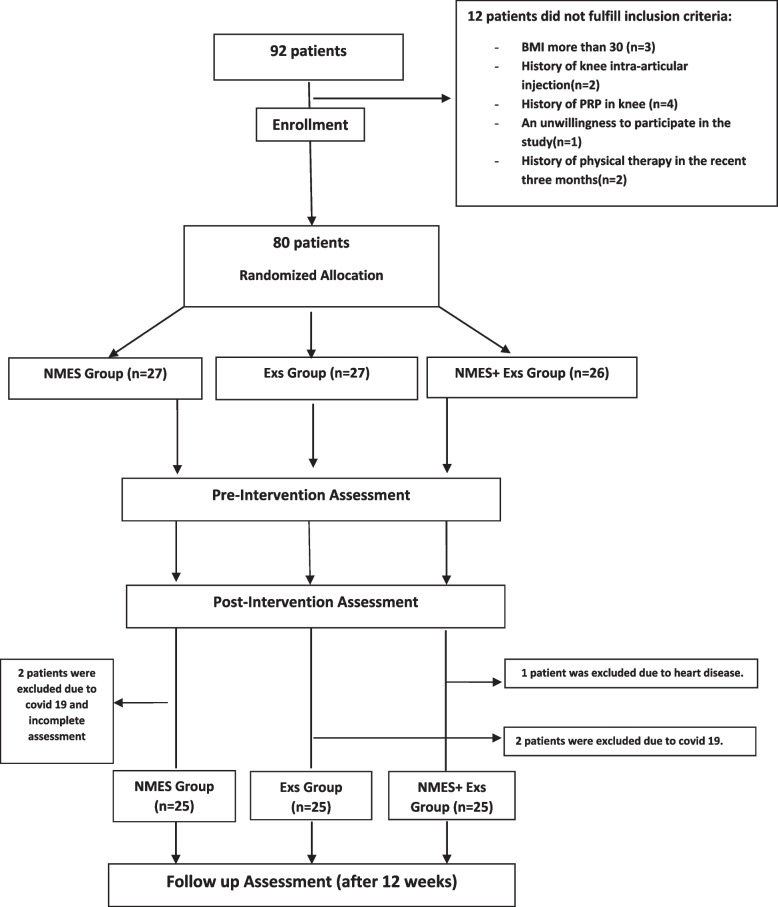
Consort flow diagram of recruitment and allocation of the participants

Initially, 92 patients were included in the study, after excluding 12 patients who did not meet the criteria, the remaining 80 individuals were randomly allocated into three groups: (1) NMES, (2) Exs, and (3) NMES + Exs using a computer-generated random allocation (Fig. 1). The assessors evaluating the patients were kept unaware of their assigned groups to ensure blinding throughout the study. The research data will be analyzed by a statistical consultant who is not privy to information regarding the patient groups.

### Interventions

All patients underwent 12 supervised treatment sessions, three times a week. The interventions were administered by the same therapist across all groups.

*NMES group:* The patients in the NMES group received IF currents exclusively to enhance the strength of their VM muscle while lying in a supine position with a knee pillow. The BTL-4825 S Topline combined electrotherapy system, which included Pre-Modulated IF two-pole currents, was used for this purpose. The position of electrodes was determined based on the alignment of QF fibers, with particular emphasis on the Rectus Femoris and VMO. The proximal electrode was aligned with the Rectus Femoris, and the distal electrode was positioned over the VMO, with its lower edge placed 3 cm above and medial to the patella [22]. The specific treatment parameters used were as follows: Pulse Width: 600μs; Carrier Frequencies = 4,000—4,050; Beat (Sweep) Frequency = 50 Hz; Treatment duration: 15 min. The current intensity (mA) was adjusted to ensure muscle contractions were comfortable and effective, avoiding any pain or discomfort.

*Exs (control) group:* The control group patients followed a supervised protocol that included five exercises. Each exercise was performed in three sets of 10 repetitions, with a ten-second hold for each exercise with a 2-min rest between sets. three times a week [11, 23]. The exercises included:

1. *Static contraction of the quadriceps femoris in a supine position.*
2. *Straight legs raising (SLR) in a supine position.*
3. *Terminal knee extension in a supine position.*
4. *Static contraction of hip adductors in a supine position.*
5. *Wall-sit in the range of 90*^*º*^* knee flexion.*

*NMES* + *Exs group:* In addition to the application of NMES, the patients in this group also followed the exercise protocol of the control group.

All patients received transcutaneous electric nerve stimulation (TENS) pre-intervention for their pain management using the Combined BTL-4825 S Topline device from the UK. TENS was applied to the patients using a frequency of 100 Hz, a pulse width ranging from 50 to 100 μs, and a quadratic biphasic symmetrical pulse shape. This treatment lasted for 20 min and involved the placement of two self-adhesive electrodes on either side of the knee. The intensity (mA) was individually adjusted to reach the threshold of a tingling sensation for each patient [4].

To ensure consistency in patients' medication, all patients were instructed to discontinue the use of non-steroidal anti-inflammatory drugs one week prior to and throughout the intervention. They were only prescribed Acetaminophen, with a maximum daily dosage of 2 g, to be used if they experienced pain.

### Outcome measures

In cases where patients experienced knee issues in both knees, the knee that exhibited more symptoms was selected for inclusion in the study.

Pain severity was assessed using a VAS, which ranged from 0 to 10. FROM was measured with a goniometer, while thigh muscle atrophy was evaluated by measuring TG using an inelastic tape at 18 cm proximal to the anteromedial knee joint line [24].

The Chison i3 4D Color Doppler Ultrasound Imaging System from China Jiangsu, CHISON Medical Technologies Co., equipped with a 10 MHz linear-array probe, was utilized to evaluate the thickness of the VMO. An investigator, unaware of the groups, performed all measurements in a relaxed mode. To measure the VMO thickness, patients were positioned in a supine position with the knee fully extended and the leg supported by sandbags on either side of the ankle to maintain a neutral hip position.

The VMO thickness was measured 4 cm superior and 3 cm medial to the superior pole of the patella [25]. To ensure optimal contact and minimize image misinterpretation, an ultrasound gel was applied between the skin and the probe. The probe was placed with gentle pressure on the skin at the specified locations to acquire clear images of the VMO. Three images of the muscle were captured and saved in JPEG format for subsequent analysis, as shown in a supplementary file. The average of the measurements from the three images was used for further analysis.

Furthermore, the physical functions of the patients were evaluated through 6MWT and TUG [4]. The secondary outcomes included assessing the patients' scores on the pain, physical function, and stiffness subscales of the Persian version of the Western Ontario and McMaster Universities Osteoarthritis Index(WOMAC), which has been culturally validated [26].

## Data analysis

The statistical analyses were performed using SPSS 26.0 software (SPSS Inc., Chicago, IL, USA). The normality of the data was assessed using the Shapiro–Wilk test, while the assumption of homogeneity of variance was examined using Levene's test. Intragroup results were analyzed using Repeated Measure ANOVA and Friedman test. To identify significant changes in the data among the groups and across the three stages of assessments, both parametric (ANOVA) and nonparametric (Kruskal–Wallis) methods were employed. Additionally, the Bonferroni, Conover, and Tukey tests were utilized to determine any between-group differences.

A significance level of *p* ≤ 0.05 was established. Moreover, in a pilot study, ten healthy subjects underwent repeated measurements with a seven-day interval to assess the intra-test reliability of objective assessments. The reliability was evaluated using interclass correlation coefficients (ICC) with a 95% confidence level. The ICC values between the first and second measurements were 0.94, 0.89, 0.92, and 0.89 for FROM, 6MWT, TUG, and the VMO thickness, respectively.

## Results

Figure 1 presents a CONSORT diagram illustrating the patient flow throughout each stage of the trial. Initially, 92 patients were screened in response to a general call. However, the study and assessments were completed by a total of 75 patients.

### Baseline characteristics of patients

Prior to the study, there were no significant differences observed between the groups in terms of the demographic variables listed in Table 1, as determined by ANOVA analysis, indicating the groups were well-matched. Moreover, no significant differences were found between the groups at baseline in terms of clinical data, including variables such as VAS, FROM, TG, 6MWT, TUG, the VMO thickness, and WOMAC scores, as shown in Table 2.

**Table 1:** Baseline demographic and clinical characteristics of the participants

	NMES Group (*n* = 25) Mean (SD)	Exs Group (*n* = 25) Mean (SD)	NMES + Exs Group (*n* = 25) Mean (SD)	*P* value *(P* ≤ *0.05)*
**Quantitative variables**	**Mean (SD)**			
Age (years)	57.31(5.80)	57.83(5.97)	58.15 (6.36)	0. 911^a^
BMI (kg/m^2^)	26.68(2.01)	25.77(2.43)	26.29 (2.48)	0. 487^a^
History of KOA (years)	4.73(3.75)	5.50(4.03)	4.88(3.80)	0. 809^a^
**Qualitative variables**	**Frequency (percentage)**			
K-L radiological stage (%)				
Grade II	15 (60%)	14(56%)	13(52%)	0.459^a^
Grade III	10(40%)	11(44%)	12(48%)	0.568^a^
Involved Knee (%)				
Right	18(72%)	19 (76%)	17(68%)	0.702^a^
Left	7(28%)	6(24%)	8(32%)	0.651^a^
Involved Joint (%)				
Patellofemoral (PFJ)	6(24%)	5(20%)	5(20%)	0.671^a^
Tibiofemoral (TFJ)	10(40%)	10(40%)	9(36%)	0.723^a^
Mixed (PFJ + TFJ)	9(36%)	10(40%)	11(44%)	0.422^a^

^a^Non-significant difference (ANOVA test)

**Table 2:** Changes in VAS, FROM, TG, VMO Thickness, 6MWT, TUG and WOMAC (total and subscales) between the groups

	NMES Group (*n* = 25) Mean (SD)	Exs Group (*n* = 25) Mean (SD)	NMES + Exs Group (*n* = 25) Mean (SD)	*P* value *(P* ≤ *0.05)*
*VAS (0–10)*				
pre-intervention	5.10(1.37)	5.17(1.01)	5.47(1.32)	0.615^c^
post-intervention	3.18(1.04)	3.66(0.97)	2.94(1.34)	0.226^c^
after 12 weeks	1.78(0.89)	2.84(0.80)	1.93(1.27)	0.022^a^
*P value*	< 0.0001^e^	< 0.0001^e^	< 0.0001^e^	
*FROM (degree)*				
pre-intervention	115.26(3.09)	110.32(3.27)	120.51(4.22)	0.79^g^
post-intervention	117.13(2.02)	110.50(2.59)	123.35(3.81)	< 0.0001^b^
after 12 weeks	118.60(2.59)	112.26(2.14)	128.50(2.25)	< 0.0001^b^
*P value*	< 0.0001^d^	0.397^f^	< 0.0001^d^	
*TG (cm)*				
pre-intervention	49.31(2.23)	48.22(2.63)	50.25(2.43)	0.316^g^
post-intervention	50.45(2.95)	47.55(2.95)	52.80(1.95)	< 0.004^b^
after 12 weeks	52.15(2.26)	48.35(2.26)	54.25(2.16)	< 0.0001^b^
*P value*	< 0.0001^d^	0.023^d^	< 0.0001^d^	
*VMO Thickness (mm)*				
pre-intervention	16.01(0.43)	15.60(0.73)	15.01(0.63)	0.361^g^
post-intervention	17.22(0.59)	15.72(0.39)	16.42(0.19)	< 0.003^b^
after 12 weeks	17.71(0.62)	15.81(0.56)	18.17(0.32)	< 0.0001^b^
*P value*	< 0.0001^d^	0.076^f^	< 0.0001^d^	
*TUG (second)*				
pre-intervention	9.71(0.68)	9.98(0.65)	9.87(0.58)	0.603^c^
post-intervention	8.60(0.70)	9.42(0.26)	8.91(0.23)	< 0.0001^a^
after 12 weeks	7.72(0.63)	9.01(0.31)	8.22(0.65)	< 0.0001^a^
*P value*	< 0.0001^e^	< 0.0001^e^	< 0.0001^e^	
*6MWT(m)*				
pre-intervention	442.36(47.14)	437.64(51.50)	432.65(54.41)	0.842^c^
post-intervention	523.19(59.05)	470.60(59.38)	511.53(45.21)	0.038^a^
after 12 weeks	548.93(36.65)	493.23(62.30)	526.20(47.70)	0.029^a^
*P value*	< 0.0001^e^	< 0.0001^e^	< 0.0001^e^	
*WOMAC pain subscale*				
pre-intervention	9.20(0.78)	9.73(0.18)	9.34(0.64)	0.688^g^
post-intervention	6.13(0.68)	6.43(0.65)	6.24(0.71)	0.061^g^
after 12 weeks	5.16(0.96)	6.56(0.98)	3.98(0.51)	
*P value*	< 0.0001^f^	< 0.0001^f^	< 0.0001^f^	0.003^b^
*WOMAC stiffness subscale*				
pre-intervention	3.84(1.17)	3.82(0.81)	3.81(0.98)	0.097^c^
post-intervention	2.94(0.85)	2.87(0.52)	2.47(0.87)	< 0.0001^a^
after 12 weeks	1.93(0.97)	2.03(0.97)	1.40(1.05)	< 0.0001^a^
*P value*	< 0.0001^e^	< 0.0001^e^	< 0.0001^e^	
*WOMAC function subscale*				
pre-intervention	33.98(2.93)	34.86(2.96)	35.25(2.57)	0.646^g^
post-intervention	27.46(2.49)	28.50(2.87)	28.32(1.92)	0.231^g^
after 12 weeks	21.43(1.32)	21.56(1.29)	22.13(1.75)	0.017^b^
*P value*	< 0.0001^f^	< 0.0001^f^	< 0.0001^f^	
*WOMAC total score*				
pre-intervention	45.96(3.32)	48.46(3.60)	46.87(4.04)	0.720^g^
post-intervention	37.53(4.12)	39.48(2.16)	38.67(3.27)	0.071^g^
after 12 weeks	26.96(1.84)	29.26(2.20)	27.73(2.98)	0.007^b^
*P value*	< 0.0001^f^	< 0.0001^f^	< 0.0001^f^	

Abbreviation: *VAS* Visual Analog Scale, *FROM* Flexion Range of Motion, *TG* Thigh Girth, *VMO* Vastus Medialis Oblique, *TUG* Timed Up and Go, *6MWT* Six-Minute Walk, *WOMAC* Western Ontario and McMaster Universities Osteoarthritis Index

^a^Significant difference post intervention among groups based on ANOVA

^b^Significant difference post intervention among groups based on Kruskal–Wallis test

^c^Non-significant difference in the baseline among groups based on ANOVA

^d^Significant difference based on Friedman test

^e^Significant difference based on repeated measure ANOVA

^f^Non-significant difference based on Friedman test

^g^Non-significant difference in the baseline based on Kruskal–Wallis test

*Intragroup comparisons-* Significant statistical differences were observed in VAS, FROM, 6MWT, TUG, the VMO thickness, and WOMAC (total and subscales) within each group during the three assessment phases, except for FROM and the VMO thickness in the Exs group, as presented in Table 2.

*Intergroup Comparison -*Post-intervention and follow-up data were analyzed using ANOVA and Kruskal–Wallis test to determine inter-group differences, and the Bonferroni, Conover, and Tukey tests were employed to assess any significant differences between the groups. The outcomes of the intergroup comparisons were presented as follows:

A significant difference in pain reduction was detected between the groups by ANOVA only after the 12-week follow-up. When comparing the VAS values after the 12-week follow-up, the Tukey test revealed a significantly greater reduction in pain in the NMES group.

The Kruskal–Wallis test revealed significant differences among the groups in TG, FROM values, and the VMO thickness after the intervention and at the 12-week follow-up. The Conover and Tukey tests demonstrated a significant increase in TG and knee flexion range, respectively, in the NMES + Exs group after the intervention and at the 12-week follow-up. Additionally, the Conover test indicated that the NMES + Exs group was superior in increasing the VMO thickness compared to the other two groups after the intervention and at the 12-week follow-up. Furthermore, ANOVA analysis found significant differences among the groups in TUG and 6MWT at both post-intervention and follow-up assessments. The Tukey test revealed that NMES was superior in both post-intervention assessments. There were significant differences among the groups in the WOMAC stiffness subscale post-intervention and after follow-up. The Tukey test affirmed that the NMES + Exs group exhibited superior results in improving the WOMAC stiffness scores of the patients following the completion of treatment and at the follow-up stage. After the intervention, no significant difference was observed among the three groups in WOMAC pain and function subscales. However, during the follow-up period, significant differences emerged in these subscales among the groups, as determined by the Kruskal–Wallis test. The Conover test further demonstrated that, at the 12-week follow-up, the NMES + Exs group exhibited superior WOMAC pain and function scores compared to the other two groups. The Kruskal–Wallis test showed no significant difference in WOMAC total score post-intervention and but there was a significant difference in total score between the groups after a 12-week follow-up. The Conover test indicated that the NMES group was superior in WOMAC total score compared to the other two groups at the 12-week follow-up. The WOMAC total score did not show any significant difference after the intervention, as indicated by the results of the Kruskal–Wallis test. But, during the follow-up period, a significant difference in the total score emerged among the groups. The Conover test further demonstrated that the NMES group achieved significantly superior WOMAC total scores compared to the other two groups at the follow-up.

## Discussion

The impairment of QF muscle function is thought to hasten the physiological deterioration associated with KOA. However, treatment options that directly address this muscle group are currently limited. In this clinical trial, the focus was on investigating the effects of NMES with IF current, exercise alone, and a combination of NMES with exercise on pain, FROM, the thickness of the VMO, WOMAC scores, and physical function in women diagnosed with primary KOA.

Our results revealed that, when comparing the three groups, the NMES group showed a more significant decrease in pain during the follow-up period. Additionally, the NMES + Exs group exhibited better results in terms of FROM, TG, and the VMO thickness after the intervention and at the 12-week follow-up. Moreover, the NMES group showed significant improvements in TUG and 6MWT at both post-intervention and follow-up assessments. We observed significant differences between the groups concerning WOMAC (total and subscales). Specifically, the NMES + Exs group demonstrated greater improvement in WOMAC stiffness scores both immediately after the intervention and during the follow-up period. At the follow-up assessment, this group outperformed the other two groups in WOMAC pain and function subscales. On the other hand, the NMES group showed better results in WOMAC total score compared to the other groups at the 12-week follow-up.

Numerous studies have highlighted the beneficial impact of low-frequency NMES in improving muscle strength [12, 13, 22, 27]. However, only a limited number of researches have explored the effectiveness of medium- frequency (IF) NMES in improving muscle function in KOA.

*Pain –* Alleviating pain in patients with KOA is of utmost importance, as it significantly impacts their performance and quality of life. One noteworthy finding from the study was a reduction in knee pain across all three groups after the intervention. However, during the follow-up assessment, the NMES group exhibited superior pain relief compared to the other groups. The initial lack of difference in pain outcomes among the groups immediately after the intervention could be attributed to the effectiveness of both NMES and exercise therapy in improving patients' conditions. It is essential not to disregard the pain control effect of TENS, as all groups used it similarly.

The NMES employed in this study utilizes a medium-frequency current to transfer electrical stimulation to nerve fibers that innervate muscles. This stimulation triggers electrical potentials in motor nerves, resulting in muscle contractions that emulate the effects of exercise. A range of analgesic processes likely contributes to these outcomes, involving mechanisms such as endogenous opioids and non-opioids, exercise-induced hypoalgesia, and the anti-inflammatory effects of exercise leading to diminished inflammation biomarkers [4, 9, 28]. Consequently, it appears that the improvements in muscle strength may underlie the observed pain reduction. The findings align with prior systematic reviews conducted by de Oliveira Melo et al., Zeng et al., and Giggins et al., which also reported pain relief in knee osteoarthritis through the utilization of NMES [29–31].

According to Laufer et al., the application of NMES (high-voltage constant current) on the QF muscle demonstrated a decrease in pain among KOA patients immediately after the intervention and during the follow-up period [32]. Similarly, Imoto et al. demonstrated a significant pain improvement with the use of NMES (50 Hz biphasic, asymmetrical pulsed current) [9]. Jin et al. studied the impact of low-frequency NMES on the VM muscle in elderly women with KOA. Consistent with the findings of our study, they observed a significant decrease in pain and concluded that utilizing NMES on the VM could be an effective approach for alleviating pain in KOA patients [33]. Sabharwal and Joshi conducted a study to examine the effects of NMES (50 Hz biphasic, asymmetrical pulsed current) compared to conventional treatment and neuromuscular exercises in patients with knee osteoarthritis (KOA). Their results, akin to our findings, demonstrated that NMES could lead to a reduction in pain [7]. Strengthening the QF, specifically focusing on the VMO, has proven effective in alleviating stress on the knee joint affected by osteoarthritis. This reduction in joint stress plays a vital role in effectively managing KOA pain [34, 35].

*FROM-* Patients with KOA experience pain and reduced activity, leading to the development of peri-articular tissue fibrosis and adaptive muscle shortening, which, in turn, restricts the ROM in the knee. The NMES + Exs group exhibited better results in terms of FROM post-intervention and at the 12-week follow-up.

The utilization of both NMES and exercise seems to have a more substantial impact on improving knee ROM. Nevertheless, it is important to acknowledge that the exercise protocol employed in the study does not specifically focus on enhancing the flexibility of tissues surrounding the joint.

During the progression of osteoarthritis, the knee ROM gradually declines. However, the combination of NMES and exercise leads to improved condition of the thigh muscle, resulting in increased patients’ ROM. Notably, the increase in ROM observed immediately after the intervention was sustained in the patients during the 12-week follow-up, representing an intriguing finding in this study.

Regrettably, most of the research in the field of NMES for patients with KOA focuses on individuals who have undergone total knee replacement. Consequently, only a limited number of studies have investigated the effects of NMES on knee range of motion in patients with knee osteoarthritis. Sabharwal and Joshi's study revealed that NMES could significantly enhance knee range of motion (ROM), which aligns with our own study findings [7].

*Thigh girth-* TG measurement is a commonly used method to evaluate the reduction in thigh muscle bulk. At both the post-intervention and 12-week follow-up, the NMES + Exs group exhibited better outcomes in TG. Unfortunately, there is limited research on the effects of exercise and NMES, particularly with medium-frequency currents, on TG in patients with KOA. Consequently, comparing the findings of this research with other studies is not feasible. Medium-frequency currents have an advantage as they can readily penetrate deep tissues, stimulating muscles due to the skin's low impedance. Additionally, they are better tolerated, offer greater comfort, and can produce significantly higher peak torque compared to low-frequency NMES [32].

*The thickness of VMO muscle*- Numerous studies have provided evidence of weakness, atrophy, and reduced cross-sectional area and the thickness of the QF muscles, particularly the VMO, in patients with KOA. Unfortunately, researchers have suggested that weakness and atrophy of the thigh muscles, especially VMO, may be the initial clinical finding even before patients report symptoms of the disease, such as pain and as the severity of the disease progresses, muscle weakness further exacerbates [33, 34]. The VMO plays a crucial role in terminal knee extension and serves as a dynamic stabilizer, preventing lateral deviation of the patella. The NMES + Exs group exhibited better results in TG both immediately after the intervention and at the 12-week follow-up. Vaz et al. conducted research to explore the effect of NMES using an 80 Hz frequency on the thickness of the Vastus Lateralis in female patients with KOA, employing sonography. They observed an increase in the thickness of the mentioned muscle, which aligns with our study's findings [35]. Likewise, Devrimsel et al. conducted a similar study, investigating the impact of NMES on the thickness of the Vastus Lateralis in patients with KOA. Their results were consistent with our current study, indicating an increase in the thickness of the vastus lateralis muscle [36].

It appears that NMES can effectively contribute to increasing muscle thickness, irrespective of the current frequency or the muscle type being investigated [12, 22]. However, the importance lies in the fact that medium-frequency interferential currents are more comfortable in inducing muscle contractions through electrical stimulation, making this method easier for patients to tolerate. Several potential factors could contribute to the increase in the thickness of the VMO muscle. These include the augmentation of active motor units, activation of thicker muscle fibers that are less engaged during regular activities, synchronization of motor unit activity within the muscle, facilitation of motor neuron function, improved synaptic facilitation, increased fiber excitability, and overall enhancement of motor control [4, 37, 38].

*Functional Tests-* The functional disability observed in individuals with KOA frequently stem from pain, muscle weakness, and muscle atrophy. Based on our research findings, the NMES group demonstrated notable improvements in the TUG and the MWT during both the post-intervention and follow-up evaluations. NMES alone appears to have effectively contributed to the improvement of patients' performance, potentially attributed to its ability to alleviate pain, and increased TG, and ROM. Our research findings align with those of Imoto et al. and Laufer et al., who also obtained similar results when evaluating the functional outcomes of their patients [9, 25]; the difference between their approach and ours lies in the use of the low-frequency current in their NMES intervention, whereas we employed a medium-frequency current.

According to previous studies, NMES induces changes in the motor recruitment process, leading to increased activation of muscle fibers, particularly type II muscles involved in intense contractions. It helps in enhancing the strength and oxidative capacity of thigh muscles in KOA patients. Moreover, NMES seems to activate afferents that facilitate sensorimotor changes within the central nervous system, leading to swift enhancements in motor control. Also, NMES promotes an increase in muscle cross-section and boosts performance, thereby resulting in improved walking speed for patients. It is worth noting that using medium-frequency NMES facilitates easier and more efficient current penetration through the skin to reach muscles and nerves [7, 12, 38].

In a prior investigation conducted by Sax Do et al., it was observed that NMES seems to have a significant impact on enhancing quadriceps muscle strength and alleviating pain in patients with KOA [36]. In a study conducted by Labanca et al., they utilized NMES with alternative biphasic waves having a frequency range of 75–85 Hz, which resulted in an observed improvement in QF muscle strength [37].

*WOMAC—*The WOMAC questionnaire is widely used by KOA patients to self-assess pain, stiffness, and function. The NMES + Exs group displayed significant improvements in WOMAC stiffness scores both immediately after the intervention and during the follow-up period. Moreover, during the follow-up assessment, this group performed better than the other groups in WOMAC pain and function subscales. Additionally, the NMES group demonstrated better results in the WOMAC total score compared to the other groups at the 12-week follow-up. After the intervention, a significant difference was observed between the groups in the WOMAC stiffness subscale scores, whereas the other scores did not show significant distinctions. This lack of significance may be due to the interventions having similar effects in improving the patient's symptoms and performance. However, a significant difference was evident in all WOMAC total and subscale scores among the groups at the 12-week follow-up.

The current study's results regarding the enhancement of WOMAC scores align with the research conducted by Vaz et al. Their findings also indicate that NMES can serve as a viable treatment option to manage symptoms and enhance the performance of patients with KOA [27].

In contrast to the findings of the current study, Durmuş et al. did not observe any significant difference in the WOMAC questionnaire scores when utilizing NMES in KOA patients [38]. Similarly, Imoto et al. also found no significant difference in the WOMAC scores after implementing NMES on the QF muscle [9]. They suggested that the absence of a significant functional disorder among their studied patients could be the reason for the lack of difference in WOMAC total and subscale scores. Nevertheless, it should be noted that the impact of NMES in controlling symptoms and improving performance may increase with the severity of functional disorders in patients.

NMES appears to contribute to the improvement of activities of daily living, pain, and physical function in the patients. Additionally, the significance of TENS in managing pain should not be overlooked within the groups. The likely reason for the improvement in WOMAC scores during the follow-up period can be attributed to a considerable reduction in pain and its persistent effect, along with the enhancement in muscle strength. KOA patients tend to avoid engaging in physical activities despite the potential benefits due to their experience with chronic pain. As a result, their mobility is often restricted to avoid pain, leading to a significant decline in joint usage, weakened muscle strength, increased joint stiffness, reduced range of motion (ROM), and diminished physical performance.

Despite the outbreak of COVID-19, the current study demonstrated a low drop-out rate, indicating that the intervention for managing KOA was well-received and considered satisfactory. Importantly, the researchers took significant measures to prioritize patient health and mitigate the risk of COVID-19 transmission. Every patient was provided with essential personal protective equipment, including masks, gloves, and face shields. Throughout the treatments and waiting periods, strict adherence to social distancing measures was maintained. Additionally, interventions were conducted in isolated spaces with stringent adherence to safety protocols, and other essential facilities were provided.

An important strength of this study is its novel approach of combining exercise and medium-frequency NMES, which is believed to have synergistic effects in reducing pain and improving muscle strength and physical function in KOA patients. Moreover, medication intake was closely monitored to avoid any potential biases that could impact the study's outcomes. Additionally, there were no reported adverse effects by the patients during or following the interventions.

However, our study does have certain limitations. One of them is the relatively small sample size, consisting mainly of non-obese female patients with grade 2 and 3 KOA. Therefore, the generalizability of our findings to other populations, including male patients with KOA, remains uncertain. Additionally, the study solely focused on medium-frequency NMES and did not compare its effectiveness with other NMES frequencies. Future research should explore the efficacy of NMES with other frequency currents and include broader patient demographics to establish more comprehensive treatment guidelines for KOA management. Furthermore, conducting additional studies that incorporate electromyographic evaluation will enhance our understanding of how NMES impacts muscle improvement.

## Conclusion

The finding supports the feasibility of the application of NMES with IF medium-frequency as a safe and well-tolerated treatment option for KOA patients. Combining NMES with exercise appears to be more effective in improving WOMAC subscales, FROM, TG, and the VMO thickness both immediately after the intervention and at the 12-week follow-up. Meanwhile, the NMES-only group showed significant enhancements in functional tests and WOMAC total score during the follow-up period.

### Supplementary Information

**Additional file 1.** The images of exercise protocol for SET and IET groups according to the explanations given in Table 1.

## Data Availability

All data relevant to the study are included in the article. The datasets generated during and/or analyzed during the current study are available from the corresponding author, Azar Moezy, upon reasonable request. Please get in touch with Azar Moezy at moezy.a@iums.ac.ir for data requests. The data will be provided in a de-identified format and subject to any necessary ethical and legal approvals. Any materials used in this study are available upon request from the corresponding author.

## Abbreviations

KOA: Knee Osteoarthritis
NMES: Neuromuscular Electrical Stimulation
QF: Quadriceps Femoris
VMO: Vastus Medialis Oblique
VAS: Visual Analog Scale
FROM: Flexion Range of Motion
TG: Thigh Girth
TUG: Timed Up and Go
6MWT: Six-Minute Walk
WOMAC: Western Ontario and McMaster Universities Osteoarthritis Index
