# Supplementary material for: A controlled randomized trial with a 12-week follow-up investigating the effects of medium-frequency neuromuscular electrical stimulation on pain, VMO thickness, and functionality in patients with knee osteoarthritis
Source: BMC Musculoskelet Disord. 2024 Feb 20;25:158. doi: 10.1186/s12891-024-07266-8 (PMC10877797; doi:10.1186/s12891-024-07266-8)
Supplement: Supplementary file 1 — Additional file 1. The images of exercise protocol for SET and IET groups according to the explanations given in Table 1. [file 12891_2024_7266_MOESM1_ESM.docx]

**The images of exercise protocol for SET and IET groups according to the explanations given in table 1.**


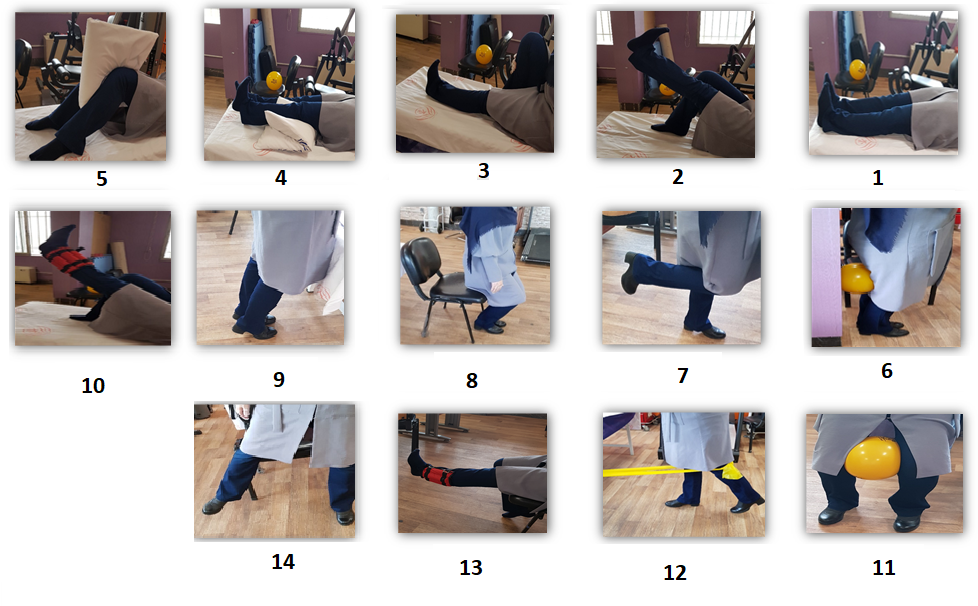
 the exercise protocol for SET and IET groups
